# Utilization of agro-industrial wastes and by-products by *Bacillus subtilis* for the biogenic synthesis and In-Depth characterization and cytotoxicity assessment of silver nanoparticles

**DOI:** 10.1186/s12866-025-03998-2

**Published:** 2025-05-14

**Authors:** Basma T. Abd-Elhalim, Salma H. Mohamed, Badawi A. Othman, Mohammed N. Abou Seada

**Affiliations:** Department of Agricultural Microbiology, Faculty of Agriculture, Ain Shams University, Hadayek Shoubra, PO Box 68, Cairo, 11241 Egypt

**Keywords:** Agro-industrial wastes, *Bacillus subtilis*, Biosynthesis, Characterization, Silver nanoparticles

## Abstract

Silver nanoparticles (AgNPs) have garnered significant attention due to their diverse applications in nanotechnology, biomedicine, and environmental science. This study explores the biogenic synthesis of AgNPs utilizing various agro-industrial wastes by *Bacillus subtilis* AMD2024 as a sustainable and eco-friendly alternative to conventional chemical synthesis methods. We evaluated a range of agro-industrial by-products, including blackstrap sugarcane molasses, sugar beet waste, and arish cheese whey, for their potential in AgNPs production. Comprehensive characterization techniques—such as X-ray diffraction (XRD), atomic absorption spectroscopy (AAS), dynamic light scattering (DLS), UV-visible spectroscopy, high-resolution scanning electron microscopy (HR-SEM), zeta potential analysis, and Fourier transform infrared spectroscopy (FTIR)—were employed to assess the properties of the synthesized nanoparticles. The DLS and UV-visible study findings were obtained at a particle size of 15.63 nm and a surface plasmon resonance (SPR) of 0.593 at 450 nm, respectively. The time course showed the maximum concentration of AgNPs after 48 h at 1.535 mg/L. The preliminary findings suggest that the size of the synthesized AgNPs was effectively reduced to as low as 4.849 nm when using blackstrap sugarcane molasses. The cytotoxicity assessment revealed a half-maximum inhibitory concentration (IC_50_) of 200 mg/mL against normal kidney epithelial cell lines, indicating the potential for safe applications in infection control and water treatment. This study highlights the applicability of agro-industrial waste valorization in nanoparticle synthesis, showcasing its promise for developing effective antimicrobial agents in various sectors.

**Supplementary Information:**

The online version contains supplementary material available at 10.1186/s12866-025-03998-2.

## Introduction

In recent decades, nanotechnology and nanoparticles—materials smaller than 100 nm—have attracted much interest. It is commonly recognized that because of their enormous surface area, great dispersion in solution, and other characteristics, nanoparticles (NPs) have a variety of remarkable physical and chemical properties. Numerous synthesis processes, such as chemical, biological, and physical methods, have been reported to be used to create NPs. Because they produce toxic chemical waste, conventional nanoparticle manufacturing methods include several physical and chemical processes that can be expensive and harmful to the environment. As a result, the need for economical, environmentally friendly, and sustainable substitutes for the traditional synthesis of metal and/or metal oxide nanoparticles has grown significantly over the past few decades [1].

The synthesis of NPs was primarily done using two methods, such as “top to bottom” and “bottom to up.” In the former, bulk material is broken down into smaller, finer nanoparticles using a range of techniques such as sputtering, laser ablation, grinding, and milling; in the latter, NPs were produced by atoms self-assembling into new nuclei that develop into nanoscale particles using chemical and biological processes [2].

In contrast to the expensive, dangerous, and toxic chemical and physical processes, the study found that biological synthesis techniques provide a simple, safe, and non-toxic alternative [3]. For instance, microorganisms have been investigated as potential biocatalysts for the synthesis of AgNPs and have demonstrated several benefits, including a high yield, nontoxic properties, and cost [4]. However, microbe-mediated AgNPs manufacturing involves microorganisms like bacteria and fungi that can transform metal ions into nanoparticles due to the presence of enzymes and other biomolecules [5]. A common site for the formation of NPs is the bacterial cell membrane, which has several functional groups that serve as ligands for metallic ions [6]. Many organic phytochemicals found in biological entities (plants and microbes) can convert Ag^+^ ions to metallic silver (Ag^◦^) nanoparticles. These include proteins, carbohydrates, polyols, phenols, terpenoids, alkaloids, amino acids, enzymes, flavonoids, glycosides, etc. Similar reduction processes can be carried out by microbial cellular and extracellular oxidoreductase enzymes [1]. AgNPs’ exceptional antidiabetic, anti-aging, antioxidant, antimicrobial, and insecticidal properties have led to widespread use in various industries, including biomedicine, drug delivery, food preservation, and agriculture [7–9]. The size, shape, and stability of AgNPs were controlled by optimizing several parameters, including temperature, reaction time, and silver nitrate concentration [10]. AgNPs induce cytotoxicity by causing oxidative stress, DNA damage, and the release of cytokines [11]. AgNPs can enter a mammal’s body through several pathways, such as skin contact, ingestion, inhalation, and intravenous injection [12]. AgNPs’ toxicity can also differ based on several variables, including their size, shape, concentration, and agglomeration or aggregation [1]. Concerning their cytotoxicity, AgNPs induce toxic effects primarily through mechanisms such as oxidative stress, DNA damage, disruption of cellular membrane integrity, and the release of pro-inflammatory cytokines [1, 12]. The ability of AgNPs to penetrate cellular membranes can lead to various harmful effects, including mitochondrial dysfunction and apoptosis, thereby elevating concerns regarding their safety in medical applications. Despite these concerns, the unique properties of AgNPs allow for widespread applications across various industries, including biomedicine, food preservation, and agriculture. For instance, their efficacy as antimicrobial agents in coatings, wound dressings, and water filtration systems underscores their potential utility in controlling infections and improving hygiene practices [50, 51, 53]. Wilson et al. [13] illustrated that numerous methods, such as atomic force microscopy (AFM), scanning electron microscopy (SEM), energy dispersive X-ray analysis (EDX), X-ray diffraction (XRD), ultraviolet-visible (UV–vis) spectrophotometry, Fourier transform infrared (FTIR) spectrometry, and particle size analysis, were used to characterize the biosynthesized AgNPs from *K. pneumoniae*.

This study introduces a novel and environmentally friendly approach to synthesizing AgNPs by utilizing *Bacillus subtilis*, a bacterium known for its non-toxic and eco-friendly characteristics, in conjunction with various agro-industrial wastes and by-products. Traditional chemical and physical methods for nanoparticle synthesis pose several drawbacks, including toxicity and environmental harm. In contrast, the biological synthesis approach using *B. subtilis* not only minimizes the impact on the environment but also enhances the biosynthesis efficiency of AgNPs.

The choice of *B. subtilis* is particularly significant because it exhibits several traits that make it a superior candidate for biogenic synthesis as follows: *B. subtilis* is recognized as a safe microorganism with a long history of use in food and agriculture, which reduces the potential health risks associated with toxic chemical synthesis methods. This bacterium possesses a range of enzymes, such as nitrate reductase, which facilitate the reduction of silver ions to form AgNPs. The presence of these enzymes makes *B. subtilis* effective in encouraging consistent and efficient nanoparticle formation. *B. subtilis* can thrive in various environmental conditions, allowing it to utilize different agro-industrial by-products as substrates for nanoparticle synthesis. This flexibility enhances the sustainability aspect of the biosynthesis process, utilizing waste materials that would otherwise contribute to pollution. Studies have shown that AgNPs synthesized through biological methods exhibit superior antibacterial activity compared to those produced through chemical processes. This can be attributed to the natural stabilizing agents and biomolecules present in the bacterial extracts that may enhance the antimicrobial effects of the nanoparticles.

By leveraging agro-industrial wastes such as blackstrap sugarcane molasses, banana peels, and arish cheese whey, this study explores the potential of these by-products not only as substrates for nanoparticle production but also as sustainable sources that contribute to waste valorization. This innovative method underscores the dual benefit of addressing waste management while simultaneously producing valuable nanoparticles. The use of agro-industrial waste is significant for several reasons, Agro-industrial waste provides an eco-friendly alternative to conventional chemical methods of nanoparticle synthesis. By repurposing waste materials that would typically contribute to environmental pollution, this approach promotes sustainability and waste valorization. Agro-industrial by-products, such as blackstrap sugarcane molasses, banana peels, sugar beet waste, and arish cheese whey, are abundant and often underutilized. These materials serve as cost-effective substrates, minimizing the overall production costs of AgNPs while contributing to circular economy principles. The presence of these biomolecules not only aids in converting silver ions into nanoparticles but also contributes to the stabilization and functionalization of the nanoparticles, enhancing their antimicrobial properties. The unique interaction between the synthesized AgNPs and the biomolecules can lead to the development of more effective antimicrobial agents. Through comprehensive characterization techniques, this research aims to highlight the effectiveness of using *B. subtilis* in synthesizing AgNPs, paving the way for future applications in various fields and fostering sustainable practices in nanoparticle production.

## Methods

### Strain collection and standard inoculum preparation

The *Bacillus subtilis* AMD2024 strain was sourced from the Agricultural Microbiology Department at the Faculty of Agriculture, Ain Shams University in Cairo, Egypt. This strain was maintained by periodic subculturing on nutrient agar medium [14] and stored at 4 °C to preserve viability until required for use. The standard* B. subtilis* inoculum was mentioned by Benson [15]. A single loop of *B. subtilis* was transferred into a sterile 250 mL conical flask containing 50 mL of nutrient broth medium. This medium was incubated at 30 °C for 24 hours with shaking at 150 rpm to promote uniform growth. After incubation, the density of the bacterial culture was estimated by performing a serial dilution. To do this, 1 mL of the cultured broth was diluted in 9 mL of sterile saline solution to create a 1:10 dilution. This process was repeated to obtain further dilutions (1:100, 1:1000, etc.). Aliquots (typically 100 µL) from appropriate dilutions (e.g., 10^^−6^ and 10^^−7^) were plated onto sterile nutrient agar plates and incubated at 30 °C for 24–48 hours. After incubation, the colonies were counted. The number of colony-forming units (CFU) per mL was calculated using the formula [14]:

1 $$CFU/mL=(Number\;of\;colonies\times dilution\;factor)/volume\;plated\;(mL)$$

For this study, the standard inoculum used for the synthesis of silver nanoparticles was prepared with a concentration of approximately 5.4 x 10^^6^ CFU/mL, which was determined from the count on the agar plates and adjusted accordingly.

### Silver nitrate precursor preparation

Weighing 0.17 g of silver nitrate (AgNO_3_), mixing it with 1000 mL of sterile double-distilled water, and then filtering the combination through an aseptic 0.22 mm filter produced a silver nitrate (AgNO_3_) solution with a concentration of 1.0 mM [16]. Aluminium foil was put over each solution and the mixture was stored in a dark environment to prevent the silver from auto-oxidizing.

### Agro-industrial waste and byproducts collection

Blackstrap sugar beet molasses, sugar beet waste, sugarcane bagasse, banana peel, and arish cheese whey were the six local agro-industrial wastes and by-products used in the AgNPs biosynthesis process. According to Table S1, these wastes and byproducts came from a variety of sources.

### Pre-utilization treatment of agro-industrial wastes and byproducts

The employed agro-industrial wastes and by-products, including sugar beet waste, banana peels, and sugarcane bagasse, were ground up and oven-dried for an entire night at 50 °C after being washed of any impurities using warm water and tap water. The dehydrated substrates were then pulverized using a lab grinder to remove large particles and achieve a size of 5.0 mm and then kept making the powdered form for future experiments [17]. After diluting blackstrap sugar beet and sugarcane molasses 1:1 with water until the acidity reached pH 4.0, they were cooked for an hour at 100 °C in a water bath and then allowed to precipitate the undesirable metal salts overnight [18]. The proteins were denatured by heating the arish cheese whey to 121 °C for 15 minutes after the pH was brought down to 4.5 with 5 N HCl. Next, a SIGMA 2–16 P centrifuge was used to centrifuge the sample for 15 minutes at 10,000 rpm. [19].

### Biosynthesis of Bs-AgNPs using standard fermentation medium (glucose broth)

The biosynthesis of AgNPs has been demonstrated, according to [20, 21]. A 250 mL Erlenmeyer flask was filled with a milliliter of *B. subtilis* inoculum, and 50 mL of glucose liquid (broth) medium was employed as a control [14]. In a shaking incubator (Shin Saeng, South Korea), the inoculated flasks were shaken at 150 rpm for 30 °C to promote the biosynthesis of AgNPs. Twelve hours and 48 hours were spent incubating the mixture under the same conditions. Then, aseptically, 50 mL of sterile 1.0 mM AgNO_3_ solution was added. The color change in the broth medium indicated that AgNPs were being produced. Following the completion of AgNPs production, the broth culture was centrifuged at 10,000 rpm for 10 minutes. The biosynthesized AgNPs were assessed by collecting cell-free supernatant [19].

### Biosynthesis of Bs-AgNPs using alternative agro-industrial wastes and byproducts

To determine which alternate wastes and by-products were best suited for the production of AgNPs using *B. subtilis*, they were all added at a comparable concentration to glucose, the fermentation medium’s main carbon source. In Giza, Egypt, the Central Laboratory of the Agriculture Research Center collected the total carbon contents of the following compounds: 5.0, 30.0, 60.0, 53.0, 62.5, and 51.0% for banana peel, arish cheese whey, blackstrap sugar beet molasses, blackstrap sugarcane molasses, sugar beet waste, and sugarcane bagasse, respectively. The carbon content of various agro-industrial wastes used in the biosynthesis of silver nanoparticles was determined using standard analytical methods as follows: The selected agro-industrial wastes, such as blackstrap sugar beet molasses, banana peel, arish cheese whey, sugar beet waste, and sugarcane bagasse, were thoroughly washed to remove impurities. After washing, the samples were dried in an oven at a controlled temperature (typically around 50 °C) until a constant weight was achieved. The dried wastes were ground into a fine powder using a laboratory grinder. To ensure uniformity in the measurements, the ground samples were sieved to eliminate larger particles, resulting in a homogenous sample size. The carbon content of the prepared samples was measured using the dry combustion method, which involves converting the organic carbon present in the samples to CO_2_. This method typically employs an instrument known as a CHN analyzer (which measures carbon, hydrogen, and nitrogen). The ground samples (usually about 0.5 grams) were placed in the combustion chamber of the CHN analyzer. The samples were then combusted at high temperatures in the presence of excess oxygen, converting all carbon into CO_2_. The amount of CO_2_ produced was measured, allowing for the calculation of the carbon content based on standard calibration curves. The carbon content for each agro-industrial waste was expressed as a percentage by weight relative to the dry sample mass [22].

### The time course of the biosynthesized Bs-AgNPs

The biosynthesized AgNPs’ time course was evaluated in order to establish the optimal incubation duration required for AgNPs synthesis. A 5 ml of the growing supernatant was collected at different incubation times every 24 hours, for 72 hours, centrifuged aseptically and the amounts of AgNPs were estimated using an atomic absorption spectrometer (AAS) (Shimadzu AA-6300, USA) [19].

### Characterization and investigation of the biosynthesized Bs-AgNPs

The first indication of Bs-AgNPs synthesis was a discernible color change. AgNPs formation was the primary focus of an experiment using UV-vis spectroscopy (JASCO Corp., V-570, USA) and the culture growth supernatant at wavelengths between 400–700 and 200 and 600 nm, respectively. The Egyptian Petroleum Research Institute (EPRI), Cairo, Egypt, used an X-ray diffractometer (Shimadzu −7000, UK) and powder XRD analysis to identify the crystalline states of AgNPs. The XRD patterns were obtained at a scanning rate of 2° per minute and 2θ from 20° to 80° with the Joint Committee on Powder Diffraction Standards (JCPDS) card No. 04–0783 for silver.

For the AgNPs suspension, the zeta potential and particle size were ascertained by dynamic light scattering (DLS) and were measured at a temperature of 25 °C. The dispersant for DLS measurements was deionized distilled water, which ensures a neutral environment for the AgNPs suspension and minimizes interference from other ions. The samples were diluted to an appropriate concentration (typically 1–5 mg/L) in deionized water to avoid multiple scattering effects and achieve optimal concentration for the DLS analysis. Each DLS measurement comprised multiple acquisitions to ensure statistical relevance, typically lasting for about 10–15 minutes per sample. The same dispersant, deionized distilled water, was employed for zeta potential measurements, which facilitates clear electrophoretic mobility assessment under the influence of an electric field. Similar to DLS, AgNPs were diluted in deionized water to reach an appropriate concentration that provides reliable readings while avoiding high particle aggregation with a count rate of 286 kHz, wavelength (λ = 1.54 nm), and viscosity 0.995cp. The analysis included several runs (typically five) to confirm the results and improve reliability. Each measurement was completed within about 5–10 minutes. Disposable capillary cells were used to measure zeta potential, ensuring the cleanliness of the setup and reducing contamination risks.

The atomic absorption spectrometer (AAS) (Shimadzu AA-6300, USA) at the Creative Egyptian Biotechnologists (CEB) company in Dokki, Giza, Egypt for estimating concentrations. Characterizing the structural morphology of AgNPs using high-resolution scanning electron microscopy (HR-SEM) (FEI Quanta FEG 250 instrument). The HR-SEM provided a resolution down to 1 nm and operated at an accelerating voltage of 5 kV to achieve high-quality imaging of nanoparticle morphology. The samples were sputter-coated with a thin layer of gold to enhance conductivity before imaging. Detecting the presence of functional groups connected to the Bs-AgNPs using Attenuated Total Reflectance and Fourier transform infrared (ATR-FTIR) (THERMO NICLOT, 50, USA). FTIR spectra were recorded in the range of 400 cm⁻^1^ to 4000 cm⁻^1^. A resolution of 4 cm⁻^1^ was achieved, which is typical for FTIR spectra to differentiate functional groups effectively. Samples were analyzed in the form of KBr pellets or as thin films.

### Cytotoxicity investigation of Bs-AgNPs

The cytotoxicity activity of AgNPs was assessed against normal ccl-81 kidney epithelial cell lines using different concentrations ranging between 1000-31.25 mg/mL at the Science Way Company in Cairo, Egypt. Following the guidelines provided by [23], after inoculating the 96-well tissue culture plate with 1x10^5^ cells/mL (100 µL/well), it was incubated at 37 °C for 24 hours to create a complete monolayer sheet. Following the formation of a confluent sheet of cells, the growth material was extracted from the 96-well microtiter plates, and the cell monolayer underwent two washing medium washes. In RPMI medium with 2% serum (maintenance medium), AgNPs were split into twofold dilutions. 0.1 mL of each dilution was then analyzed in several wells. We looked at the cells for physical signs of toxicity, including granulation, rounding, shrinkage, and partial or complete loss of the monolayer. Each well was filled with 20 microliters of MTT (3-(4,5-dimethylthiazol-2-yl-)−2,5 diphenyltetrazolium bromide) solution following the preparation of the solution (5 mg/ml in phosphate buffer solution) (Bio basic Canada Inc.). To properly absorb the MTT, media were placed on a shaking table and shaken for five minutes at 150 rpm. To enable the MTT to metabolize, incubate at 37 °C with 5% CO_2_ for four hours. If necessary, take out the media and use paper towels to wipe out any remaining material from the plate. One can re-dissolve formazan, a metabolic product of MTT, in 200 µL of DMSO. Put on a shaking table and shook for five minutes at 150 rpm to mix the formazan and solvent. Optical density was obtained at 560 nm and subtracted background at 620 nm. Optical density should be directly correlated with cell quantity.

2 $$\%Cell\;viability=\frac{Mean\;Abs\;control-Mean\;Abs\;Bs.\;AgNPs}{Mean\;Abs\;control}\times100$$

where: Abs absorbance at 560 nm.

### Sample size determination and data transformations

#### Sample size determination

The sample sizes used in the experiments were determined based on a power analysis conducted before the experiments. This analysis aimed to ensure that the sample size was sufficient to detect statistically significant differences between groups with a desired power level (commonly set at 0.80) and an alpha level (commonly set at 0.05). In typical experimental setups, each treatment group in cytotoxicity tests (e.g., different concentrations of AgNPs) was repeated across at least three independent biological replicates. This means that multiple separate experiments were conducted, with each repeat providing a new dataset for analysis.

### Data transformations

Before analysis, data were assessed for normality using tests such as the Shapiro-Wilk test or visual inspection through Q-Q plots. If the data did not meet normality assumptions, a suitable transformation was applied. Common transformations, if applicable, included logarithmic transformation (log10) or square-root transformation to stabilize variance across groups. The specific transformation applied would depend on the distribution of data points once assessed. After ensuring normality, the final datasets were analyzed using two-way ANOVA, which allowed for the examination of the impact of two different factors (e.g., AgNPs concentration and exposure time) on cell viability.

### Analysis of data

The results were statistically analyzed and presented as means using the IBM® SPSS® Statistics program (2017). 0.05 was the A *P*-value for Duncan’s test [24]. Graph Pad Prism 8.4.1 (GraphPad Software, San Diego, CA, www.graphpad.com) was used to test the difference between the groups using two-way ANOVA, and the interaction was determined to be significant as *P*<0.05. Two-way ANOVA was used to compare means across groups while accounting for interactions between factors. It provided insights into how different concentrations of AgNPs and varying exposure durations affected cell viability. Duncan’s Test: Following a significant ANOVA result, Duncan’s test was performed as a post-hoc analysis to determine which specific groups differed from one another while controlling for Type I errors. The cytotoxicity results, which were computed as IC_50_, were presented as mean ±SD. Every experiment had three participants.

## Results

### Biosynthesis of Bs-AgNPs using glucose broth medium

#### Color change observation

**Fig. 1:**
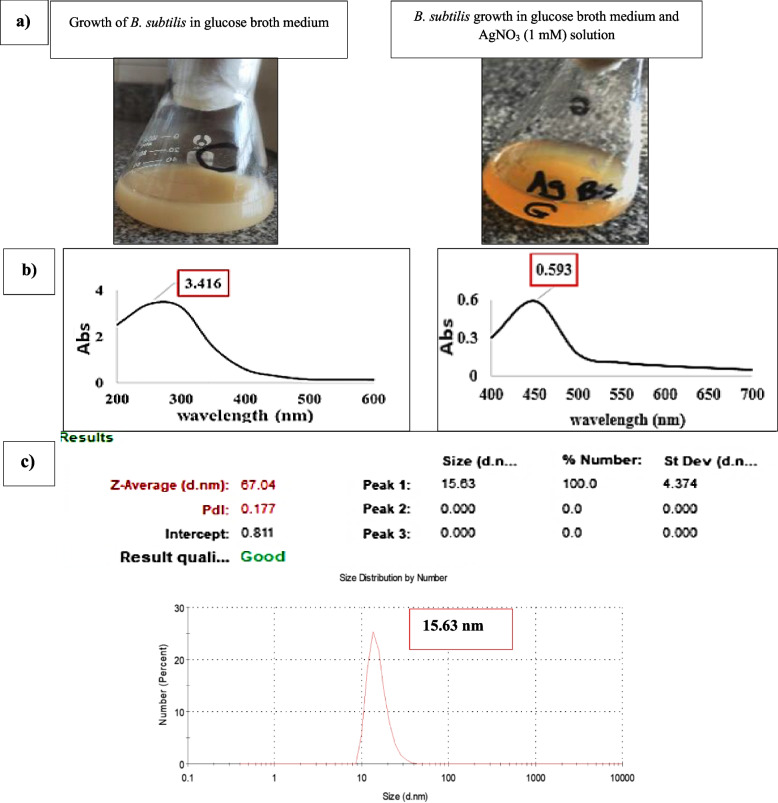
**a** The reaction color of the mixture changed from cream to pale brown, indicating the synthesis of Bs-AgNPs, (**b**) The spectra of SPR at 3.416, and (**c**) DLS characterization of AgNPs production employing shaking flasks at 150 rpm after 48 hours at 30 °C

The color of the reaction between *B. subtilis* and 1.0 mM AgNO_3_ solution changed from off-white to pale brown after 48 hours of incubation at 30 °C and shaking at 150 rpm, as shown in (Fig. 1a).

### UV-visible spectroscopy and DLS characterization

Figure 1b shows the results of the analysis of the culture supernatant at wavelengths between 200 and 600 nm using UV-vis spectroscopy compared with Bs-AgNPs solution. The culture supernatant exhibited an absorbance peak of 3.416, at λ max 250 nm, while the Bs-AgNPs reaction mixture exhibited an absorbance peak of 0.593, at λ max 450 nm. The DLS analysis data in (Fig. 1c) showed that the biosynthesized AgNPs had a size of 15.63 nm with a polydispersity index (PDI) of 0.177.

### The impact of various agro-industrial wastes and byproducts on Bs-AgNPs biosynthesis

**Fig. 2:**
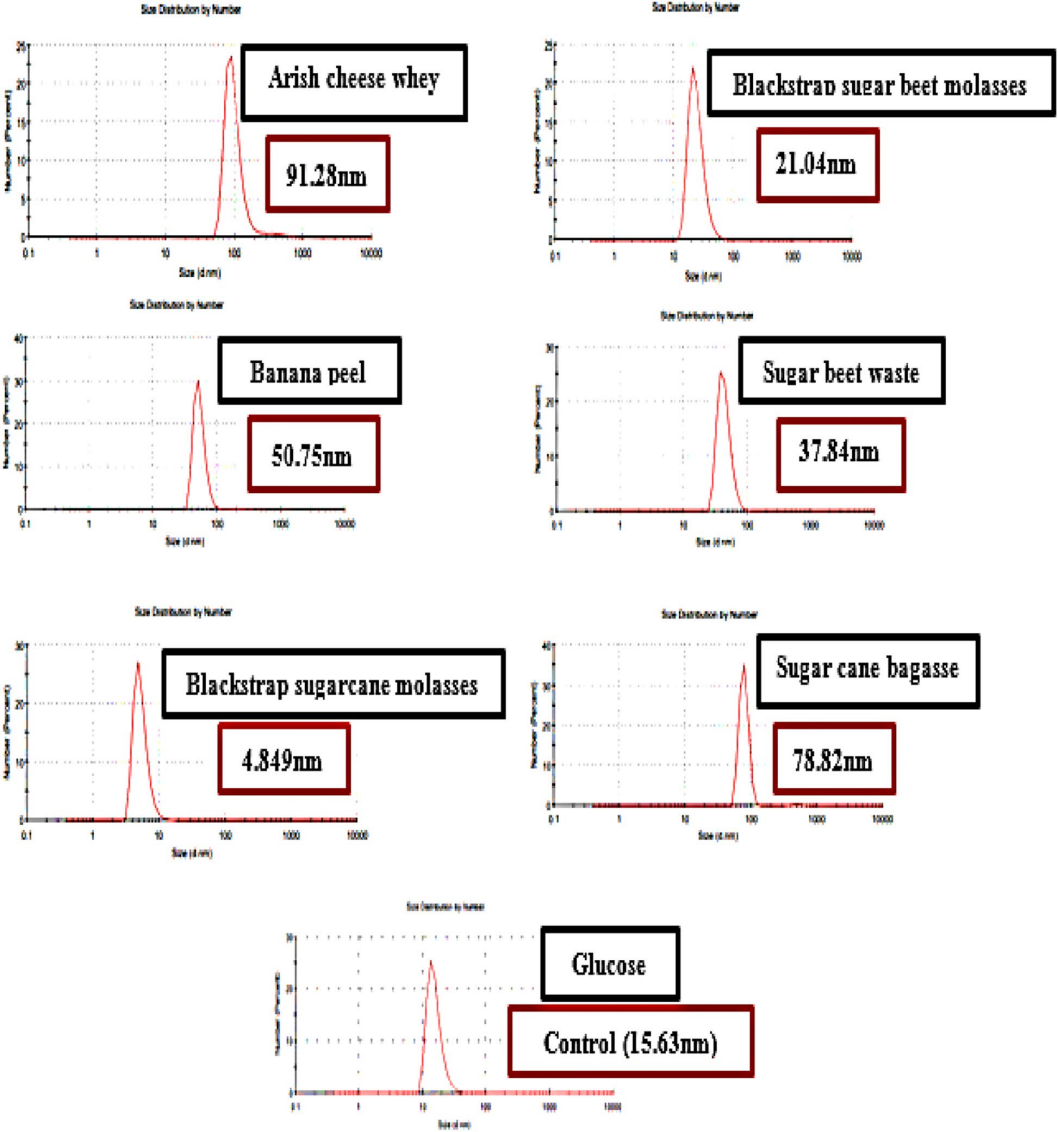
Biosynthesized Bs-AgNPs diameter size, influenced by different agro-industrial wastes and byproducts, characterized by dynamic light scattering (DLS) using shaking flasks at 150 rpm after 48 hours at 30 °C

The DLS characterization for size investigation of the biosynthesized Bs-AgNPs demonstrated the value of the agro-industrial wastes and byproducts in the biosynthesis process (Fig. 2). 68.06, 105.7, 3.122, 50.75, 50.75, and 91.28 nm were the relative diameters of the Bs-AgNPs containing arish cheese whey, banana peel, blackstrap sugar cane molasses, blackstrap sugar beet molasses, sugar beet waste, and sugarcane bagasse, respectively.

### The time course of Bs-AgNPs biosynthesis

**Fig. 3:**
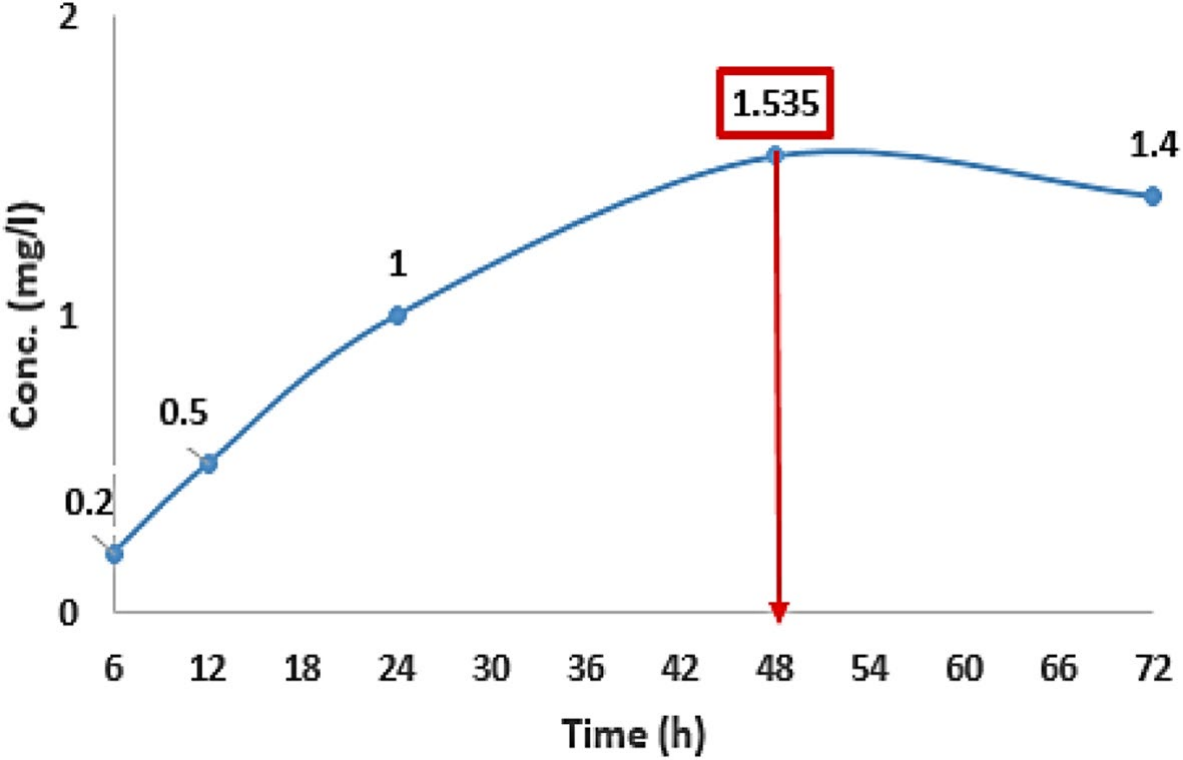
Bs-AgNPs biosynthesis time course utilizing blackstrap sugarcane molasses at 30 °C in shaking flasks spinning at 150 rpm

By calculating the concentration of the biosynthesized AgNPs in the cell-free extract, the ideal biosynthesis time course for Bs-AgNPs was determined. The optimal reaction time to get a high concentration of Bs-AgNPs was 48 hours, as shown in (Fig. 3).

### Characterization of the biosynthesized Bs-AgNPs using blackstrap sugarcane molasses, the best agro-industrial waste

#### HR-SEM analysis

**Fig. 4:**
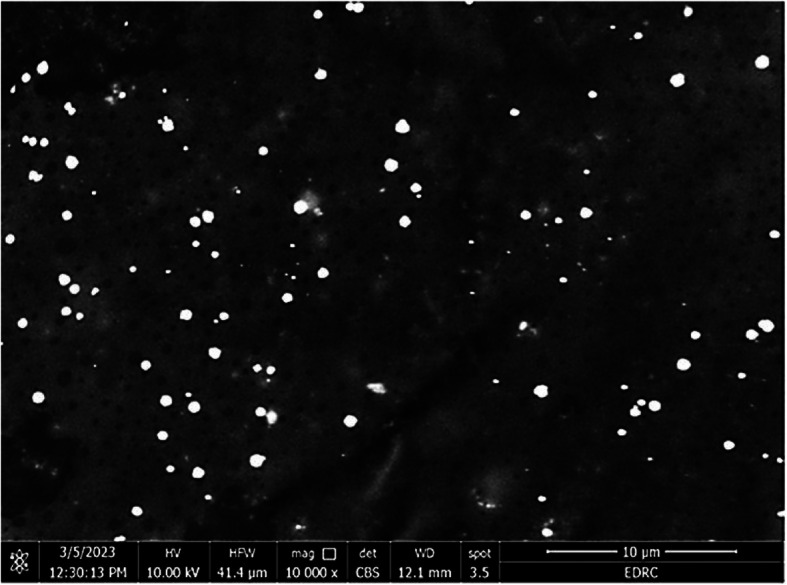
Examination of Bs-AgNPs using a high-resolution scanning electron microscope

The morphology of the Bs-AgNPs was investigated using an HR-SEM. Figure 4 illustrates the good desperate behavior of Bs-AgNPs, which are sphere particles.

#### XRD investigation

**Fig. 5:**
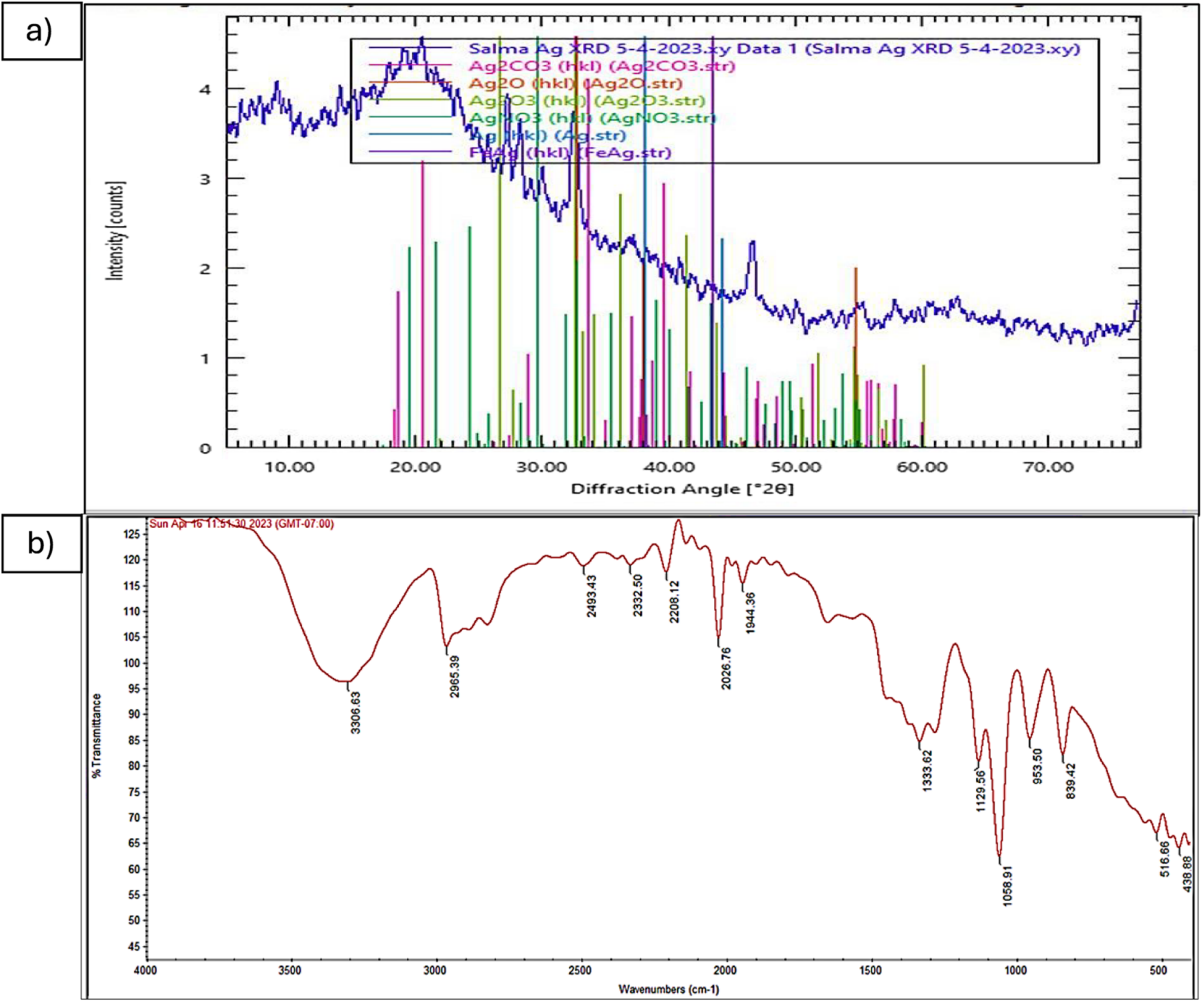
**a** XRD characterization of Bs-AgNPs, (**b**) FTIR investigation employing blackstrap sugarcane molasses after 48 hours at 30 °C using shaking flasks at 150 rpm to characterize the active chemicals that reduce, coat, and stabilize Bs-AgNPs

As seen in (Fig. 5a), the XRD analysis’s findings showed that the Bs-AgNPs’ XRD pattern contained a mixture of six compounds, including Ag_2_CO_3_, Ag_2_O, Ag_2_O_3_, AgNO_3_, and FeAg.

### ATR-FTIR characterization

The functional group presence in the cell-free extract of Bs-AgNPs ranged from 400 to 4000 cm^−1^, as illustrated in (Fig. 5b). The range of peak sites was 438.88–3306.63 cm^−1^. It was determined that the active functional groups were N-H stretching, C-H stretching, O=C=O stretching, C-C stretching, C=C=N bending, C=C=C bending, O-H stretching, C-N stretching, C-O, and C=C stretching. These groups correspond to aliphatic primary amine, alkane, carbon dioxide, alkyne, ketenimine, allene, alcohol, amine, primary alcohol, and alkene, respectively. According to the FTIR results, proteins that act as a capping agent for NPs were responsible for the reduction of AgNO_3_ to AgNPs.

### Zeta potential analysis for Bs-AgNPs

**Fig. 6:**
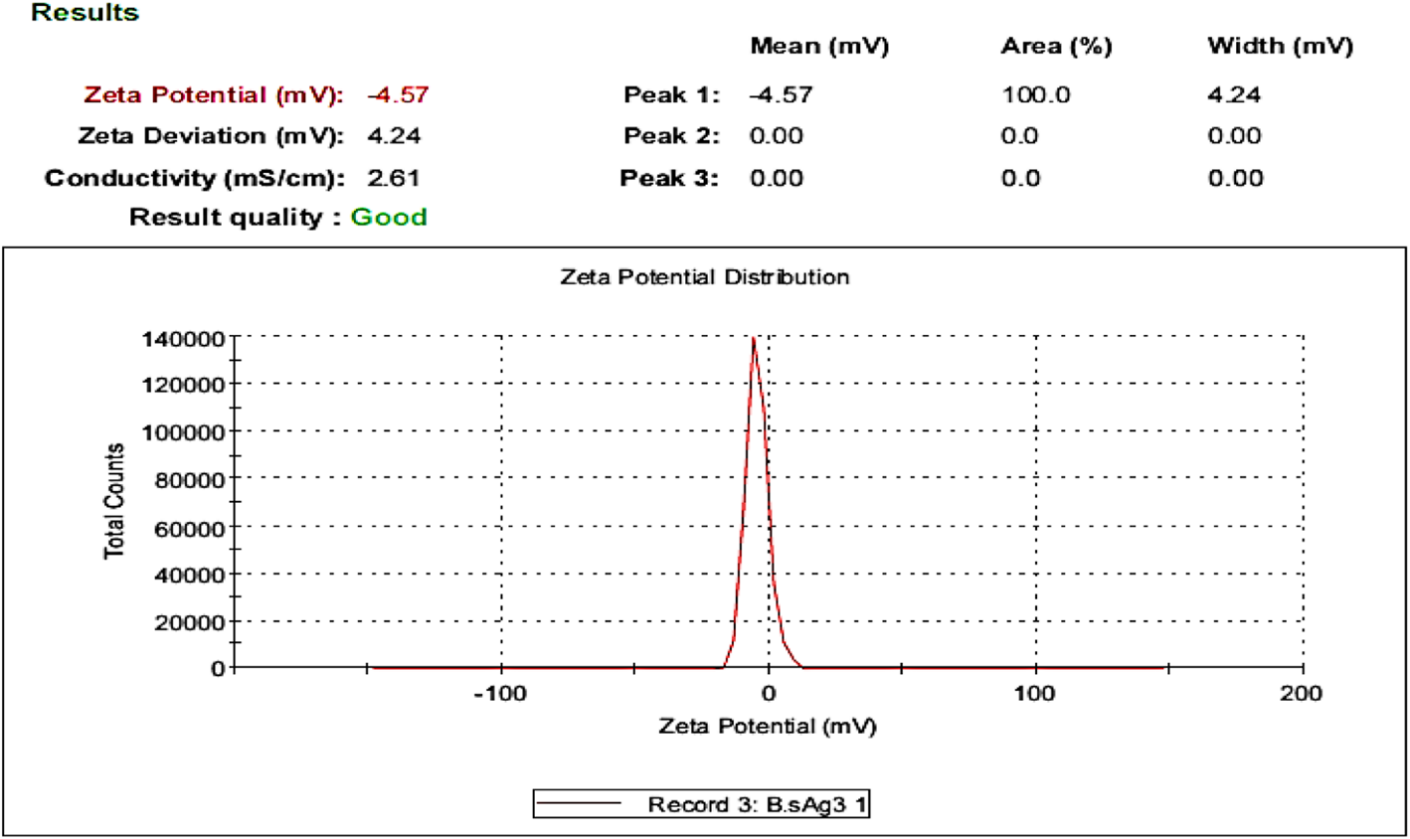
Zeta potential study of Bs-AgNPs using blackstrap sugarcane molasses in shaking flasks at 150 rpm after 48 hours at 30 °C

The investigation of the zeta potential (ξ) value for Bs-AgNPs revealed that it was −4.57 mv, as illustrated in Fig. 6.

### Determination of cytotoxicity for Bs-AgNPs

**Fig. 7:**
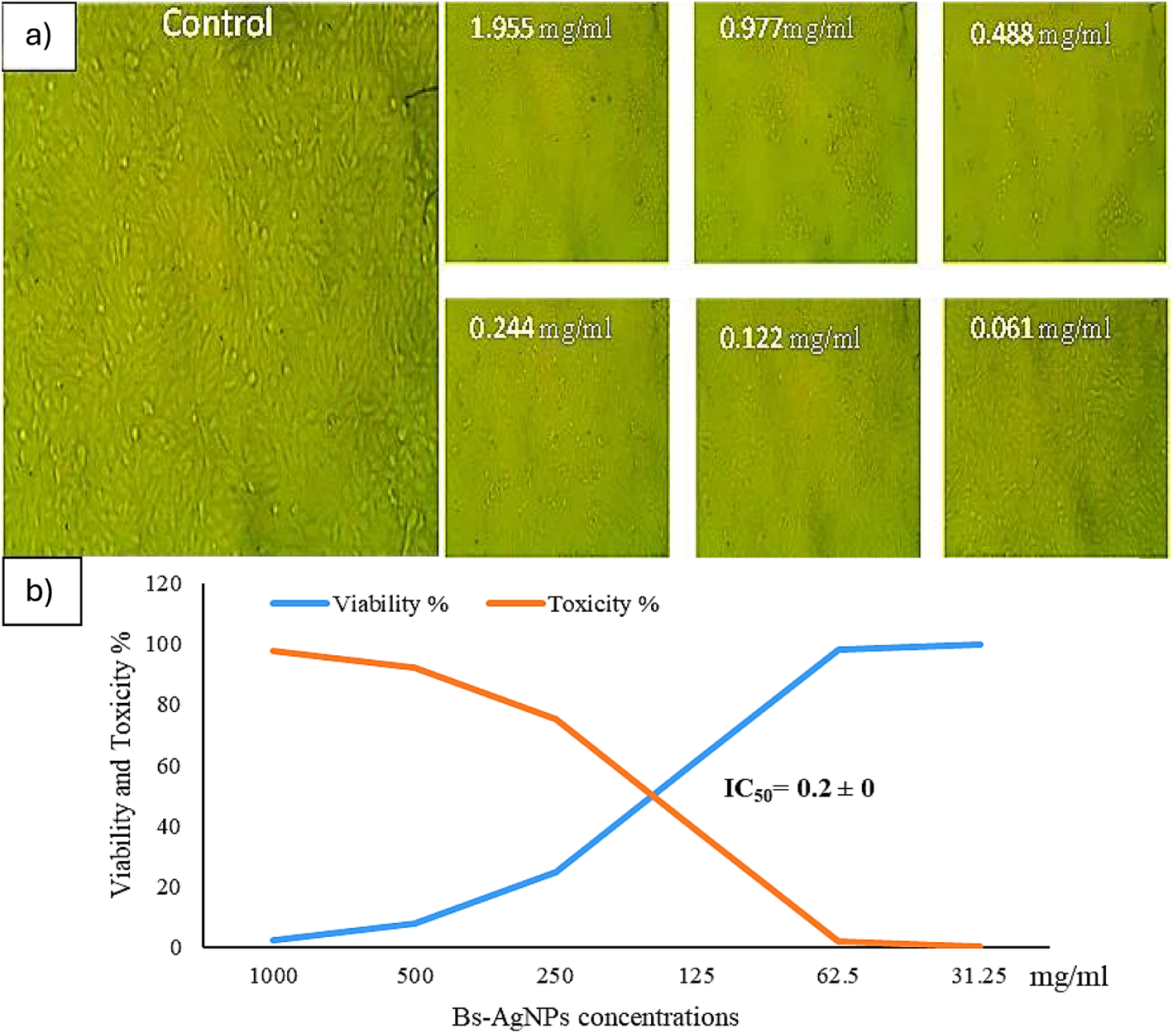
**a** The morphological changes in ccl-81 cell lines, and (**b**) The IC50 of Bs-AgNPs on ccl-81 cell lines.

As shown in Table S2, the cytotoxicity results showed that when exposed to different concentrations of Bs-AgNPs (1.955, 0.977, 0.488, 0.244, 0.122, and 0.061 mg/mL), the vitality of the ccl-81 cell lines was 3.56, 4.15, 4.5, 29.4, 86.5, and 98.37%, respectively. Fig. 7a shows microscopic images of the morphological changes in ccl-81 cell lines exposed to 1.955, 0.977, and 0.488 mg/mL of Bs-AgNPs. They used the curve to calculate the half-maximal inhibitory concentration (IC_50_) values, which measure the concentration of a pharmacological dosage required for 50% of cell death. The Bs-AgNPs added to ccl-81 cell lines had an IC_50_ value of 0.2 mg/mL, according to the findings displayed in Fig. 7b.

## Discussion

The use of nanoparticles in biomedical applications has garnered considerable attention due to their unique physical and chemical properties, which confer remarkable antimicrobial effects. The biosynthesis of NPs utilizing environmentally friendly methods, such as through the activity of microorganisms or plant extracts, has emerged as a promising avenue for producing these nanomaterials sustainably [25–29]. The ecologically benign aspect of the green synthesis of AgNPs utilizing *B. subtilis* AMD2024 makes it a unique biosynthesis strategy when compared to physical and chemical procedures. The mixture’s reaction to AgNO_3_ in combination with *B. subtilis* cell-free extract changed color, confirming the green production process of Bs-AgNPs. The production of AgNPs employing *B. subtilis* PP273436, in contrast, changed the hue from yellow to brown, according to Abdulrazzaq and Abas [30]. Using *B. zanthoxyli* GBE11 ON340757, [10] noticed a similar color shift. The findings of the UV-vis spectroscopy of the culture supernatant in comparison to the Bs-AgNPs solution indicated the culture supernatant showed an absorbance peak of 3.416 at λ max 250 nm, and at λ max 450 nm, the Bs-AgNPs reaction mixture showed an absorbance peak of 0.593. The obtained results were in close agreement with the findings of Alfryyan et al. [31] using *B. cereus* at 450 nm for AgNPs formation. In a study by Muchanyereyi et al. [32], the high absorbance utilizing plant extracts was approximately 420 nm. According to [33], the spectra of AgNPs produced by the bioreduction activity of the bacterial extract *B. subtilis* CBPPR1 revealed an absorption peak at 445 nm which is very close to the recent findings.

Proteins and enzymes like nitrate reductase in the supernatant are crucial for lowering silver ions, claims [34, 35], who investigated the impact of endophytic *A. baumannii* and *P. aeruginosa* secondary metabolites, particularly the phenolic compounds as antioxidant agents, on the production of AgNPs from silver nitrate solution, also validated this. The bioconversion of silver ions to nanoparticles in the reaction mixture was strongly indicated by the strength of the acquired SPR peak, which was scored at the optimal wavelength, thereby guaranteeing the biosynthesis process [36, 37].

Particle size of the biosynthesized AgNPs varies depending on the green synthesized procedures and the biosynthesizing conditions (temperature, shaking speed, incubation length, and metabolites for reduction of AgNO_3_). Using *B. subtilis*, the particle size of Bs-AgNPs was 15.63 nm after 48 hours at 30 °C and 150 rpm shaking flask. In the same manner, *B. subtilis* and *E. coli* produced AgNPs with an average size of 11.10 and 38.89 nm, respectively, according to Mbagwu et al. [38]. However, according to [39], the average size of AgNPs produced by *B. amyloliquefaciens* was 11.10 nm.

With a size reduction of 68.97% to 4.849 nm, blackstrap sugarcane molasses was the best byproduct and carbon source. With a size increase of 82.87% using Bs-AgNPs, the waste from arish cheese whey, on the other hand, had the largest particle size, measuring 91.28 nm. Abd-Elhalim et al. [21] found that blackstrap sugarcane molasses, which had a size of 80.5 nm, was the most appropriate carbon source for the formation of CuNPs using *P. silesiensis* strain A3, as opposed to glucose, which had a size of 87.1 nm.

Furthermore, as reported by Plaza et al. [40], brewery liquor and molasses inoculated with *B. subtilis* T-1cell-free supernatant were the most effective biosynthetic sources for the production of AgNPs. Similarly, Sherien et al. [41] showed that *Chaetomium globosum* can produce zinc nanoparticles from a range of agro-industrial wastes, such as leftover olive cake and apple, carrot, and potato peels.

Different agro-industrial wastes contain varying compositions of organic compounds, including carbohydrates, proteins, amino acids, and phytochemicals, which can influence the growth of *B. subtilis* leading to the biosynthesis and stabilization of the nanoparticles process. The amount of sugars, vitamins, and other growth elements that are essential for the synthesis of proteins, secondary metabolites, enzymes, and microbes may be the reason for the high quality of blackstrap sugarcane molasses [40]. In contrast, waste with a higher fiber or starch content might lead to larger aggregated nanoparticles due to inadequate stabilization.

By looking at the time course of the green-produced Bs-AgNPs, it was found that 48 hours of incubation was the best amount of time to achieve the appropriate concentration. The concentration of the biosynthesized AgNPs did not increase after this period. The reaction concluded at the eighteenth hour of incubation, according to the UV-Vis spectra absorbance for 1.0 mM silver nitrate combined with cell-free supernatant, and the creation of silver nanoparticles is unaffected by longer incubation times using *B. subtilis*, as demonstrated by [42]. It took a maximum of 16 hours for a combination of *Lactobacillus* and *Bacillus* species to finish fabricating AgNPs. *Bacillus* sp. alone can finish manufacturing in a maximum of 40 hours, while *Lactobacillus* sp. alone can do it in more than 40 hours [43]. According to [44], nanoparticle formation takes place during a specific period, after which the concentration and particle size stay constant due to non-agglomeration. The time course data support these findings.

Using HR-SEM, the shape and form of Bs-AgNPs were investigated. The nanosphere-shaped biosynthesized Bs-AgNPs in this work are coated with components of the free extract from *B. subtilis* cells. In the study of [41], sphere-shaped AgNPs were exposed using *B. cereus* cell-free extract. Another work by [45] used green tea leaf extract to fabricate spherical and quasi-spherical AgNPs.

According to the work headed by [37], the JCPDS cards No. 04–0783 are crucial for identifying phases in XRD analysis. Each phase has a unique card number associated with its peak positions and intensities. For AgNPs, the observed peaks in the XRD pattern should be compared against the corresponding JCPDS card to confirm the presence of silver and the absence of undesired compounds. An XRD pattern displaying peaks at 2θ values of approximately 38.12°, 44.23°, and 64.32° for silver nanoparticles suggests the presence of the (111), (200), and (220) crystallographic planes, respectively. These peaks when agreeing with the JCPDS support the identification of the synthesized nanoparticles as metallic silver. For the current results, the XRD analysis showed that the sample was not well-crystallized and free of AgNO_3_ impurities and that there was no total reduction of Ag^+^ to AgNPs. Six compounds, including Ag_2_CO_3_, Ag_2_O, Ag_2_O_3_, AgNO_3_, and FeAg, were mixed in the Bs-AgNPs’ XRD pattern, according to the results of the XRD investigation. Aswini et al. [46] demonstrated that the metallic NPs’ face-centered cubic structure’s crystalline planes were represented by the XRD peaks at 2θ values of 27.7950, 32.2943, 46.3023, 54.8490, 57.5382, 67.4964, 74.4280, and 76.7858 cm^−1^. One possible indication that silver ions are present in the generated AgNPs is the strongest peak, which is located at 32.2943 cm^−1^.

There could be many strategies to minimize the presence of additional phases such as optimization of synthesis conditions as adjusting the concentration of the silver precursor (AgNO₃) and the reducing agents can lead to a more controlled synthesis process, potentially minimizing the formation of undesired products. For instance, lower concentrations might reduce the likelihood of side reactions. Tweaking reaction parameters such as temperature and reaction time can also optimize the conditions for producing pure AgNPs. Shortening the time or controlling the temperature may prevent the formation of multiple phases.

FTIR analysis was used to examine the functional groups in the Bs-AgNPs cell-free extract, which ranged from 400 to 4000 cm^−1^. Peak positions ranged from 438.88 to 3306.63 cm^−1^. It was demonstrated that the following functional groups were active: aliphatic primary amine, alkane, carbon dioxide, alkyne, ketenimine, allene, alcohol, amine, primary alcohol, and alkene, in that order. The active functional groups were demonstrated to be N-H stretching, C-H stretching, O=C=O stretching, C≡C stretching, C=C=N bending, C=C=C bending, O-H stretching, C-N stretching, C-O, and C=C stretching. According to the results of FTIR analysis, proteins in particular were in charge of capping and reduction, whilst other components, such as fatty acids, were in charge of stabilizing utilizing *Ulvophyte* sp. MBIC10591 and *Coelastrella aeroterrestrica* (Strain BA_Chlo4), respectively [47, 48]. The present study’s functional groups (N=C=S, O-H, N-H, and C-H) are identical to those in the *B. coagulans *GBI-30, 6068 extract, with a few exceptions in other functional groups [49].

The XRD results suggest a strong molecular connection between Bs-AgNPs and *B. subtilis* development in blackstrap sugarcane molasses. Proteins like protein coating and the enzyme Ag reductase, which are responsible for the reduction and stability of AgNPs, are present when the FTIR analysis indicates bending amide and amine stretching functional groups [21].

Due to the electrostatic repulsion between charged particles, zeta potential is an essential concept for the stability of NP suspensions. Bs-AgNPs’ zeta potential analysis showed a value of −4.57 mV, according to Liu et al. [50].

In general, a zeta potential value of ±30 mV or higher is considered stable, while values below this threshold may indicate a risk of instability and agglomeration over time [2, 6]. This indicates that the biosynthesized AgNPs using *B. subtilis* were moderately stable and had a nonionic character in the cell-free supernatant of the capping molecules. AgNPs zeta potential analysis revealed a negative charge for *Rhodococcus* sp., *Brevundimonas* sp., and *Bacillus* sp., respectively, with values of 32.8, 29.6, and 28.1 mV [51].

DLS analysis gives a comprehensive characteristic of the homogeneity of the NPs in colloidal solutions by evaluating the polydispersity index (PDI) values. If the PDI score is greater or less than 0.4, the homogeneity of the NPs solution is raised; if the PDI result is equal to or greater than 1, the NPs is termed heterogeneous. According to the current findings, the biosynthesized AgNPs’ PDI score was 0.177 indicating good homogeneity [7].

Many studies have shown a wide range of IC_50_ values for AgNPs depending on various factors such as the synthesis method, size, shape, and biological source of the nanoparticles, as well as the type of cell lines used.

The cytotoxicity (IC_50_) of Bs-AgNPs on ccl-81 cells was determined to be 0.2 mg/mL (200 µg/mL). In previous studies, the cytotoxicity effect of AgNO_3_ and Ag-NPs on normal Vero cells (African green monkey kidney) produced by *B. cereus* showed IC_50_ values of 304.8 and 290.2 μg/mL, respectively, according to Alsharif et al. [52]. In another work, the IC_50_ value of AgNPs biofabricated by *Juglans regia* extract on normal (NOF18 cells) was 93.3 μg/mL, according to Ghabban et al. [2]. For renal normal cell lines originating from Vero cells, the bacterially produced Ag-NPs utilizing *B. amyloliquefaciens* had an IC_50_ value of 383.7±3.1 µg/mL, according to [54, 55].

The safety of nanoparticles often depends on the application. For biomedical applications, IC_50_ values that exceed 100 μg/mL can sometimes be considered within a “safe” range, indicating lower cytotoxicity against normal cells [2]. If the observed IC_50_ value from the current study aligns with or exceeds this threshold, it signifies that the AgNPs may be safer for potential therapeutic applications, such as drug delivery or antimicrobial use. The effectiveness of AgNPs is also critical in medical and environmental applications. Low cytotoxicity can be beneficial for applications like wound dressings, water purification, or as antibacterial agents in food packaging. Comparing the study’s IC_50_ results with other formulations helps in determining whether these nanoparticles possess a favorable balance of potency (antimicrobial activity) and safety (cell viability) for potential therapeutic and industrial applications. So it is essential to approach the use of these AgNPs with caution in therapeutic contexts. Exploring mechanisms to reduce cytotoxicity and carefully considering dosing regimens will be essential steps in optimizing their application while minimizing the risk of adverse effects. Understanding how these nanoparticles interact with biological systems will continue to be a significant area of research aimed at enhancing both safety and efficacy in biomedical applications.

## Conclusion

In conclusion, this study contributes to the growing body of knowledge on environmentally friendly methods for nanoparticle synthesis and underscores the potential of utilizing agro-industrial waste materials. Future work will focus on optimizing the synthesis conditions and expanding the scope of applications for the biosynthesized silver nanoparticles. This study explored the biosynthesis of silver nanoparticles (AgNPs) using *Bacillus subtilis* AMD2024 and various agro-industrial wastes. The findings indicate that the biological synthesis method employed can effectively produce AgNPs with favorable characteristics, including an average particle size of approximately 15.63 nm and a peak in absorbance at 450 nm. The characterization of these nanoparticles through X-ray diffraction (XRD), atomic absorption spectroscopy (AAS), dynamic light scattering (DLS), UV-visible spectroscopy, high-resolution scanning electron microscopy (HR-SEM), and Fourier transform infrared spectroscopy (FTIR) provided valuable insights into their structural and morphological properties. A half maximum inhibitory dose (IC_50_) of 200 mg/mL against normal kidney epithelial cell lines was found during the cytotoxicity assessment, suggesting the possibility of safe uses in water treatment and infection prevention. The application of agro-industrial waste valorization in nanoparticle synthesis is demonstrated in this work, indicating its potential for creating potent antimicrobial agents across a range of industries.

## Supplementary Information

Supplementary Material 1.

## Data Availability

“The datasets generated and analyzed during the current study are available in the name [Bacillus subtilis strain AMD2024] repository, [https://www.ncbi.nlm.nih.gov/nuccore/PP527379].”
