# Supplementary material for: Utilization of agro-industrial wastes and by-products by Bacillus subtilis for the biogenic synthesis and In-Depth characterization and cytotoxicity assessment of silver nanoparticles
Source: BMC Microbiol. 2025 May 14;25:291. doi: 10.1186/s12866-025-03998-2 (PMC12080011; doi:10.1186/s12866-025-03998-2)
Supplement: Supplementary file 1 — Supplementary Material 1. [file 12866_2025_3998_MOESM1_ESM.pdf]

Supplementary file

Table S1: Collection of agro-industrial wastes and by-products sources.

Agro-industrial wastes and by-products	Source
1- Arish cheese whey	Local market, Shoubra Al-Khaimah, Cairo.
2- Banana peel	Local market, Shoubra Al-Khaimah, Cairo.
3- Blackstrap sugar beet molasses	Delta sugar company, Kafr El sheikh.
4- Blackstrap sugarcane molasses	Sugar refinery factory, El-Hawamdia.
5- Sugar beet waste	Delta sugar company, Kafr El sheikh.
6- Sugarcane bagasse	Local sugar cane juice shop, Shoubra Al-Khaimah, Cairo.

Table. (S2): Cytotoxicity activity of Bs-AgNPs with various concentrations against ccl-81 cell line

ID	mg/ml	O.D			Mean O.D	±SE	Viability %	Toxicity %	IC50 ± SD
vero	--------	0.733	0.742	0.739	0.738	0.002646	100	0	mg
Bs-AgNPs	1.955	0.024	0.03	0.025	0.026333	0.001856	3.568202349	96.43179765	0.2 ± 0
	0.977	0.031	0.029	0.032	0.030667	0.000882	4.155374887	95.84462511	
	0.488	0.03	0.036	0.034	0.033333	0.001764	4.516711834	95.48328817	
	0.244	0.217	0.228	0.207	0.217333	0.006064	29.44896116	70.55103884	
	0.122	0.645	0.632	0.64	0.639	0.003786	86.58536585	13.41463415	
	0.061	0.726	0.73	0.722	0.726	0.002309	98.37398374	1.62601626	
